# Pseudoaneurysm of the left ventricular free wall occurring after cardiac surgery of endocarditis affecting mitral and aortic valves: a case report

**DOI:** 10.1186/s43044-023-00334-9

**Published:** 2023-01-24

**Authors:** Chighaly El Hadj Sidi, Varha Isselmou, Mohamed Feissal Mohamed Ahmed, El Hadj Abdelaziz Diop, Taleb Ekhyar Argueina, Haimouda Mady, Khaled Boye

**Affiliations:** 1 Department of Cardiovascular and Thoracic Surgery, National Center of Cardiology, Nouakchott, Mauritania; 2 Department of Cardiology, National Center of Cardiology, Nouakchott, Mauritania; 3 Department of Cardiothoracic Anesthesiology, National Center of Cardiology, Nouakchott, Mauritania

**Keywords:** Endocarditis, *Staphylococcus* hominis, Left ventricle, Pseudoaneurysm, Case report

## Abstract

**Background:**

Infective endocarditis remains a serious condition. Left ventricular pseudoaneurysm may complicate the clinical course of infective endocarditis or occur postoperatively.

**Case presentation:**

We describe a case of a pseudoaneurysm of the left ventricular lateral wall which developed one month following cardiac surgery of active endocarditis affecting aortic and mitral valves. The diagnosis was established by transthoracic echocardiography and computed tomography angiography of the chest. Urgent cardiac surgery is performed with excision of the pseudoaneurysm and direct closure of the defect. The patient had a complete recovery and was discharged on the twelfth postoperative day.

**Conclusions:**

Left ventricular pseudoaneurysms are rare but potentially fatal. The symptoms revealing such complications are very diverse. Surgical treatment can be offered to younger patients.

**Supplementary Information:**

The online version contains supplementary material available at 10.1186/s43044-023-00334-9.

## Background

Infective endocarditis is a serious disease that causes several local complications, such as perivalvular extension with the formation of abscesses, fistulas, and pseudoaneurysms [1]. In the absence of statistics, it is not possible to obtain an accurate estimate of the incidence of infective endocarditis in the general population in Africa. A few studies have shown that endocarditis accounts for less than 0.5% of adult cardiovascular admissions [2]. Left ventricular pseudoaneurysms is very uncommon in infective endocarditis, usually presenting in cases of myocardial infarction [3]. Their formation after infective endocarditis accounting for less than 1% of all left ventricular pseudoaneurysms, and are fatal with a 35–40% risk of rupture [4, 5]. These pseudoaneurysms result from incomplete rupture of the left ventricular wall contained by adherent pericardium and/or scar tissue [4, 6]. Thus, unlike a true left ventricular aneurysm, a left ventricular pseudoaneurysm contains neither endocardium nor myocardium [4]. The formation of left ventricular pseudoaneurysms due to infective endocarditis occurs by three mechanisms: septic coronary embolism, dissemination from an adjacent perivalvular abscess, and seeding of the endocardium by a regurgitant jet [1, 7]. These pseudoaneurysms have a strong tendency to grow rapidly, which increases the risk of their rupture and consequent pericardial tamponade which is a fatal condition [8, 9]. So, in most cases, it is considered a diagnostic and therapeutic emergency. In this case report, we present a case of a left ventricular pseudoaneurysm complicating the surgical management of mitral and aortic endocarditis due to Staphylococcus hominis and successfully treated by surgical excision.

## Case presentation

We report a case of a 21-year-old young African man with a non-specific medical history, who presented with right hemiparesis along with a fever of two weeks duration. During clinical examination, we found good oral hygiene, a temperature of 37.5°c, a heart rate of 105 bpm, a blood pressure of 114/41 mmHg, a Glasgow Score of 15/15, a diastolic murmur at the aortic area of intensity 4/6 on the auscultation of the heart, a paresis and hypoesthesia of the right side of the body, and there were no skin lesions. An electrocardiogram showed sinus rhythm at 108 bpm with evidence of left ventricular hypertrophy. Routine laboratory tests showed: microcytic hypochromic anemia with hemoglobin at 8.6 g/dL, leukocytes at 7500/µL, creatinine at 100 µmol/L, and C-reactive protein at 73 mg/L. Initial diagnosis suspected infective endocarditis complicated by septic emboli leading to ischemic stroke. Indeed, computed tomography of the brain showed an acute left temporal lobe stroke of ischemic origin and transthoracic echocardiography confirmed the diagnosis of infective endocarditis by revealing the existence of several vegetations on aortic cusps with severe aortic regurgitation, dilatation of the left ventricle with ejection fraction at 57%, moderate mitral and tricuspid regurgitation with pulmonary arterial pressures at 60 mmHg. Further oral, ear, nose and throat (ENT) examinations were performed to search for the source of infection also abdominal ultrasound performed as part of the extension assessment but no specific findings were observed. Blood cultures revealed negative results. The patient was hospitalized, and an empirical combination of antibiotic therapy with Ampicillin and Gentamicin has been prescribed for him.

The clinical course was marked by the persistence of a subfebrile state and the blood cultures were repeated and returned, this time, positives for Staphylococcus hominins. Antibiotic therapy was switched to Vancomycin, and the clinical course was good with a resolution of fever and complete recovery of the sensory-motor deficit in the right side of the body. Despite two weeks of vancomycin therapy, transthoracic echocardiography showed extension of valve damage with increasing vegetations that had become mobile and the development of multiple periannular aortic abscesses along with an anterior mitral leaflet abscess. Urgent surgical intervention was performed for appropriate and early management.

**Fig. 1:**
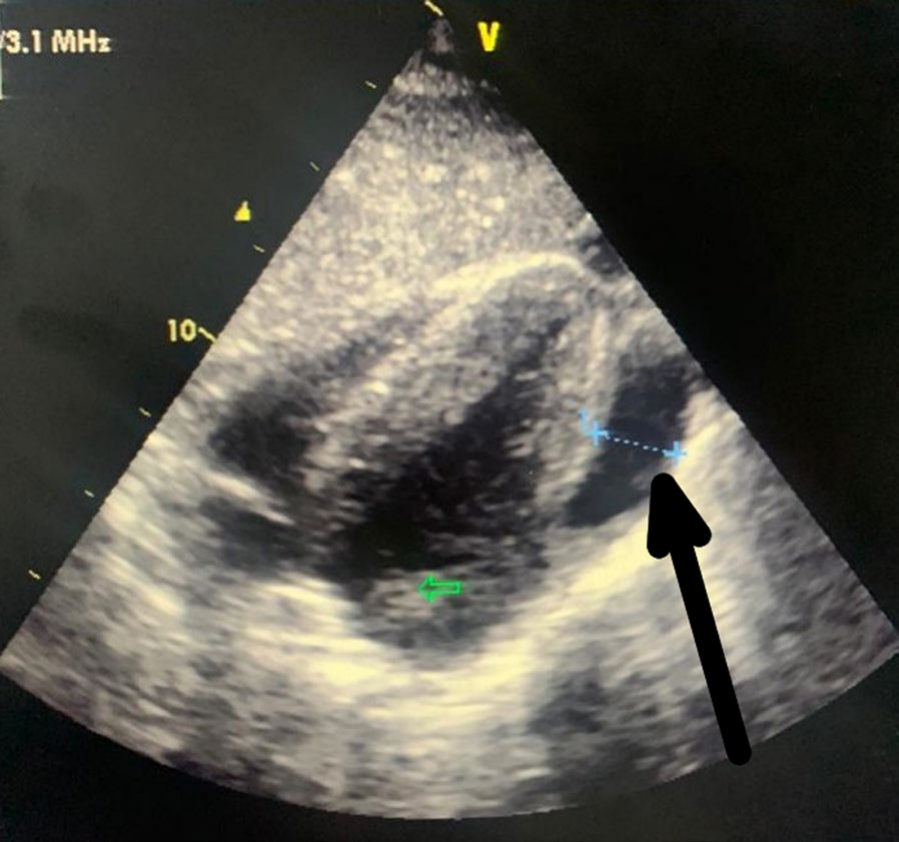
Transthoracic echocardiography showing the pseudoaneurysm of the left ventricular free wall (arrow)

**Fig. 2:**
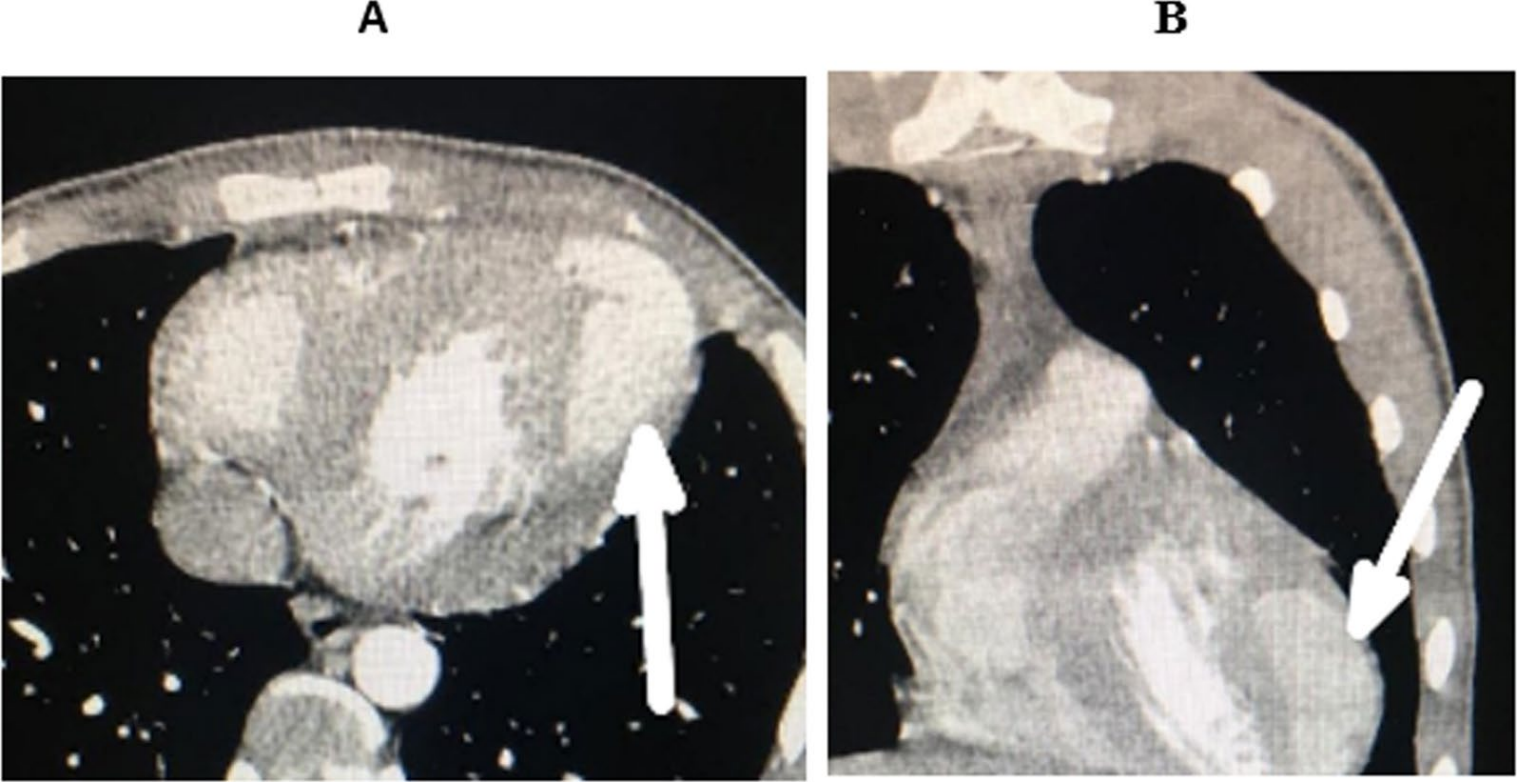
Computed tomography angiography of the chest showing the pseudoaneurysm of the left ventricular free wall (arrow). **a** axial view. **b** coronal view

**Fig. 3:**
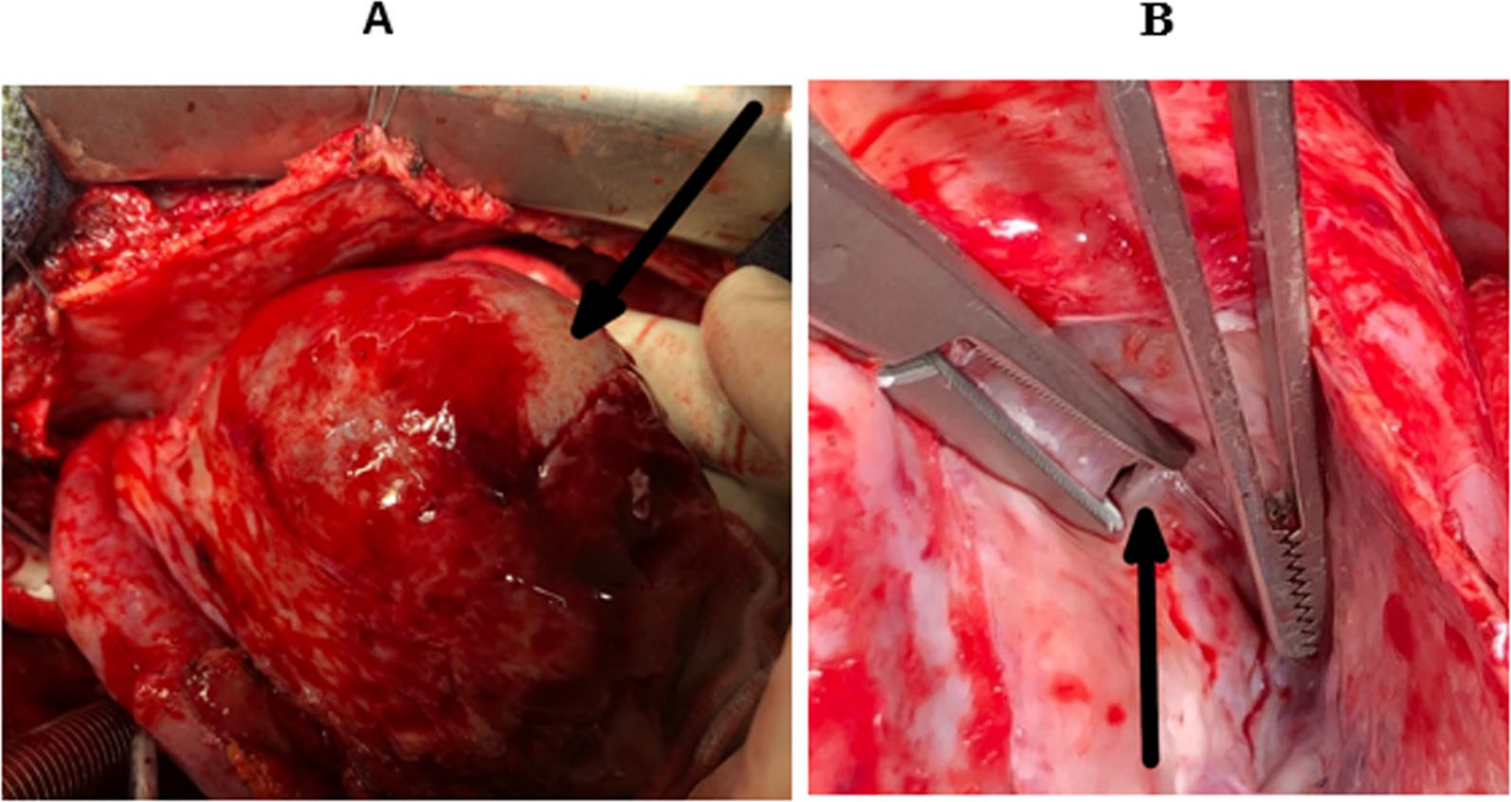
Intraoperative view showing the pseudoaneurysm of the left ventricular free wall. **a** After installation of the CPB and exposure of the pseudoaneurysm, the aneurysm sac still intact (arrow). **b** After opening the aneurysm sac, the neck of the pseudoaneurysm is opened using dissecting forceps (arrow)

The surgery was performed under cardiopulmonary bypass with aortic clamping and heart arrest. Left heart decompression was performed by a venting cannula introduced inside the left ventricle through the upper right pulmonary vein. Myocardial protection was provided by a warm-blood cardioplegia administered intermittently by the coronary ostia. Assessment of valve damage noticed a thickened bicuspid aortic valve with large vegetations and several periannular abscesses, a mitral valve with an abscess in the body of its anterior leaflet (A3), and a tricuspid valve with dilatation of its annulus. We proceeded to clean the abscess of the anterior mitral leaflet with resection of the valve tissue that surrounded it with a repair of the leaflet with an autologous pericardial patch measuring almost 2 cm in diameter and mitral annuloplasty according to Carpentier's method, resection of aortic cusps and vegetations with debridement of the periannular abscesses and valve replacement by a double-wing prosthesis and tricuspid annuloplasty according to the De Vega's method. The valve culture was positive for the same germ and we, therefore, decided to continue the antibiotic treatment with Vancomycin for six weeks (in the hospital) from the day of the intervention. The patient had a favorable recovery course initially with the obtainment of definitive apyrexia and normalization of inflammatory markers. Transthoracic echocardiography, on the tenth postoperative day, was satisfactory. But, on the thirtieth postoperative day, the patient presented with palpitation and repetitive electrocardiograms showed fleeting arrhythmias such as accelerated junctional rhythm and ventricular extrasystole (bigeminy) with a return to a basal sinus rhythm between attacks. Transthoracic echocardiography (Fig. 1 and additional file 1) and computed tomography angiography of the chest (Fig. 2) revealed a pseudoaneurysm developed from the left ventricular lateral wall measuring 67 × 43 × 26 mm with a neck of 8 mm in diameter. After one week of patient preparation (stopping the oral anticoagulant and switching on heparin), he was operated on under cardiopulmonary bypass with aortic clamping and heart arrest. Warm-blood cardioplegia is delivered through the aortic root. The pseudoaneurysm was excised (Additional file 2) and the defect on the left ventricular wall was closed by U-shaped stitches reinforced with a Teflon pledget with non-absorbable monofilament (Fig. 3). Postoperative recovery was, this time, complete, and the patient was discharged on the twelfth postoperative day. The timeline of the patient's course is given in Table 1.

**Table 1:** Timeline of the patient’s course

D 14 (two weeks before)	Fever and deterioration of the general condition
D 0 (day of hospitalization)	Ischemic stroke
	Computed tomography of the brain: acute left temporal lobe stroke
D 1	Transthoracic echocardiography: several vegetations on aortic cusps with severe aortic regurgitation
	Blood cultures: negatives
	Start of antibiotic therapy (Ampicillin-Gentamicin)
D 5	oral examination: normal
	ENT examination: normal
D 6	Abdominal ultrasound: normal
D 7	Blood cultures: positives (Staphylococcus hominis)
D 12	Start of Vancomycin and stop of Ampicillin
D 14	Stop of Gentamicin
D 25	Transthoracic echocardiography: mobile vegetations and several periannular aortic abscesses and an abscess of the anterior mitral leaflet
D 28	Surgery: aortic valve replacement, mitral valvuloplasty and tricuspid annuloplasty
D 38	Transthoracic echocardiography: good
D 59	Cardiac arrhythmias
D 60	Transthoracic echocardiography and computed tomography angiography of the chest: left ventricular pseudoaneurysm
D 66	Surgery: excision of the left ventricular pseudoaneurysm and direct closure
D 79	Stop Vancomycin
	Patient discharge

## Discussion

Unlike a true left ventricular aneurysm, the wall of the pseudoaneurysm does not contain endocardium nor myocardium. It is an incomplete rupture of the left ventricular wall enclosed by adherent pericardium and/or scar tissue [4, 6]. The outer layer of a true left ventricular aneurysm has the usual three tunics with intact [10] but thinned heart wall.

Left ventricular pseudoaneurysms are rare and potentially fatal. Their true incidence is unknown [11]. They are caused by myocardial infarction in most cases [4], while they are iatrogenic following previous cardiac surgery in 33% of cases [11]. Surgical procedures frequently associated with pseudoaneurysms include mitral valve replacement, aortic valve replacement, and correction of congenital heart disease [12]. For some authors, an infection is responsible in only 13% of cases [13].

In the presence of infectious disease, three mechanisms have been described for the formation of left ventricular pseudoaneurysms: septic coronary embolism leading to myocardial infarction and secondary rupture, dissemination from an adjacent perivalvular abscess, and seeding of the endocardium by a regurgitant jet [1, 7]. In our case, the pseudoaneurysm certainly developed in the postoperative course of valve surgery, but in a situation of an active infection, the exact mechanisms are not clear and can be common. Seeding of the left ventricular wall from an aortic perivalvular abscess or regurgitant jet that has escaped the operating team intraoperatively may be the likely cause, but iatrogenic origin cannot also be excluded. We believe that the left heart decompression cannula may have played a role in the development of the pseudoaneurysm in our patient. Its introduction through the right upper pulmonary vein and/or its replacement in the left ventricle after completion of the mitral valve repair for purging the left heart with its resulting tickling of an inflammatory endocardium can induce minimal wall trauma which progressed silently.

Regarding the clinical presentation, the revealing symptoms of pseudoaneurysm are variable and are not specific. The most common symptoms are chest pain and dyspnea [10]. Arrhythmias like junctional changeover as in our presentation are described in the literature [14]. On the other hand, the incidental discovery following transthoracic echocardiography is possible, and the proportion of asymptomatic patients varies between 10 and 48% according to studies [10]. In addition, the to-and-fro murmur which is the classic result of the physical examination is not constant (absent in our patient) and may be indistinguishable from an associated mitral regurgitation [4].

Although ventriculography remains the gold standard for the diagnosis of pseudoaneurysms [10], currently, with the advance of noninvasive techniques namely echocardiography despite its low sensitivity to detect pseudoaneurysms [15], computed tomography angiography of the chest and cardiac magnetic resonance imaging, this invasive examination has lost much of its indications. These noninvasive examinations can also guide management by suggesting the nature of the anomaly detected. If cardiac magnetic resonance imaging appears to be more helpful, its unavailability in case of emergency and its contraindication in many patients limit its use [14].

Current literature reports that 30–45% of ventricular pseudoaneurysms rupture within the first year, and therefore most authors have supported surgery as their appropriate treatment [4]. Surgical repair is the standard approach to treat left ventricular pseudoaneurysms with a reported mortality rate of 23% [4, 8]. The choice between direct or patch closure depends on the age of the pseudoaneurysm, the size of its neck, and its location. In our case, we were able to suture directly the defect, despite it being recent because it was narrow and the quality of the myocardial tissue surrounding it was good. However, a conservative approach may be recommended for asymptomatic patients and those with a higher risk of postoperative morbidity and mortality [10]. Recently, percutaneous closure has been reported as an alternative for high-risk surgical candidates [11, 16].

## Conclusions

Left ventricular pseudoaneurysms are rare but potentially fatal. They can complicate the clinical course of infective endocarditis or occur postoperatively. Regular checks by echocardiography of patients at risk of developing such a complication will help to establish the diagnosis on time. Surgical treatment can be proposed for younger patients who do not have many comorbidities.

## Supplementary Information

**Additional file 1: Video 1**. Pseudoaneurysm appearance in echocardiography.

**Additional file 2: Video 2**. Appearance of pseudoaneurysm neck after resection of the aneurysm sac.

## Data Availability

No datasets were generated or analyzed during the current study.
